# Bioinformatic Analysis of Actin-Binding Proteins in the Nucleolus During Heat Shock

**DOI:** 10.3390/genes15121580

**Published:** 2024-12-09

**Authors:** Shinya Taniguchi, Takeru Torii, Toshiyuki Goto, Kohei Takeuchi, Rine Katsumi, Mako Sumida, Sunmin Lee, Wataru Sugimoto, Masaya Gessho, Katsuhiko Itoh, Hiroaki Hirata, Junji Kawakami, Daisuke Miyoshi, Keiko Kawauchi

**Affiliations:** 1 Faculty of Frontiers of Innovative Research in Science and Technology (FIRST), Konan University, Kobe 650-0047, Japan; shinya.taniguchi.ngi@gmail.com (S.T.); grignard.tor2@gmail.com (T.T.); takeuchikohei0816@gmail.com (K.T.); rinekatsumi9@gmail.com (R.K.); nicomako0531@gmail.com (M.S.); sunminlee240216@gmail.com (S.L.); los.wrrrwve.56s2@gmail.com (W.S.); m.gessho@gmail.com (M.G.); itokatsuuu@gmail.com (K.I.); kawakami@konan-u.ac.jp (J.K.); miyoshi@konan-u.ac.jp (D.M.); 2 Graduate School of Science, Technology and Innovation, Kobe University, Kobe 650-0047, Japan; mukonosou.gotou.1219@gmail.com; 3 Department of Applied Bioscience, Kanazawa Institute of Technology, Hakusan 924-0838, Japan; hirata@neptune.kanazawa-it.ac.jp

**Keywords:** actin, nucleolus, heat shock protein 70, liquid–liquid phase separation

## Abstract

Background/Objectives: Actin plays a crucial role not only in the cytoplasm, but also in the nucleus, influencing various cellular behaviors, including cell migration and gene expression. Recent studies reveal that nuclear actin dynamics is altered by cellular stresses, such as DNA damage; however, the effect of heat shock on nuclear actin dynamics, particularly in the nucleolus, remains unclear. This study aims to elucidate the contribution of nucleolar actin to cellular responses under heat shock conditions. Methods: Nuclear actin dynamics in response to heat shock were investigated using nAC-GFP, a GFP-tagged actin chromobody, to visualize nuclear actin in HeLa cells. Bioinformatic analyses were also performed. Results: Heat shock induced the reversible assembly of nAC-GFP in the nucleolus, with disassembly occurring upon recovery in a heat shock protein (Hsp) 70-dependent manner. Because the nucleolus, formed via liquid–liquid phase separation (LLPS), sequesters misfolded proteins under heat shock to prevent irreversible aggregation, we hypothesized that nucleolar actin-binding proteins might also be sequestered in a similar manner. Using several databases, we identified 47 actin-binding proteins localized in the nucleolus and determined the proportion of intrinsically disordered regions (IDRs) known to promote LLPS. Our analysis revealed that many of these 47 proteins exhibited high levels of IDRs. Conclusions: The findings from our bioinformatics analysis and further cellular studies may help elucidate new roles for actin in the heat shock response.

## 1. Introduction

Actin, one of the most abundantly expressed proteins in cells, plays a crucial role in cellular processes such as cell migration, proliferation, and differentiation, not only through the organization of cytoskeletal dynamics, but also by regulating signaling pathways, gene expression, and chromatin remodeling [1,2,3,4]. In its adenosine triphosphate (ATP)-bound form, globular actin (G-actin) polymerizes into filamentous actin (F-actin) via nucleators such as Arp2/3, Spire, and Formins [5]. This process is regulated by Rho guanosine triphosphatases (GTPases) including Rho, Rac, and Cdc42. During polymerization, ATP-bound actin is converted to adenosine diphosphate (ADP)-bound actin, which destabilizes F-actin and promotes its depolymerization [6]. Actin depolymerization was further promoted by the actin depolymerizing factor (ADF)/cofilin and gelsolin [7].

Actin is transported from the nucleus to the cytoplasm by exportin-6 and vice-versa by importin-9 [4,8]. Many actin regulators, including nucleators and destabilizers, shuttle between compartments. However, most research on actin dynamics has focused on the cytoplasm, as detecting nuclear actin filaments is challenging because of the expected differences in the amount and structure of F-actin between the cytoplasm and nucleus [9,10]. With the development of advanced probes for visualizing nuclear actin, recent studies have increasingly focused on actin dynamics within the nucleus [10].

In the nucleus, both G-actin and F-actin are involved in chromatin remodeling and the regulation of transcription factors and RNA polymerases [2]. The formation of nuclear actin assembly is induced by various cellular stresses, such as DNA damage, viral infections, and heat shock, reflecting the growing understanding of nuclear actin dynamics in response to stress [3,4,11,12,13]. Actin–cofilin rods are known to be unstainable with fluorescent phalloidin because cofilin occupies the phalloidin-binding sites on actin [11]. These rods are formed in the cytoplasm and nucleus when actin filaments and cofilin aggregate respond to oxidative stress and heat shock [11,14]. While cofilin typically severs and depolymerizes actin filaments, it also binds to actin and promotes the formation of rod-like structures under these conditions. In contrast, DNA damage-induced nuclear actin filaments were distinct from actin–cofilin rods [12,15].

The nucleolus is a membraneless organelle formed through liquid–liquid phase separation (LLPS), which is essential for ribosomal RNA (rRNA) biogenesis [16]. Typically, cells have 1–5 nucleoli, and their shape and size are irregular and varied, respectively. Nucleolar enlargement is frequently observed in highly malignant cancers and serves as a marker of poor prognosis. Actin facilitates rRNA transcription by interacting with RNA polymerase I and transcription factors [2], such as upstream binding transcription factor (UBTF) and transcription initiation factor IA (TIF-IA). Treatment with the genotoxic agent methyl methanesulfonate (MMS) promotes the formation of actin filaments in the nucleolus [12]. However, when the formation of nuclear actin filaments is promoted by various stressors, including DNA damage, they are predominantly observed in the nucleoplasm [9]. The molecular mechanisms by which actin and its regulatory proteins localize and assemble in the nucleolus remain largely unknown.

Heat shock induced by elevated temperatures lead to protein misfolding and the disruption of normal cellular functions [17]. One of the primary effects of heat shock is the rapid suppression of rRNA synthesis [18,19], which allows cells to focus on stress recovery, because ribosome biogenesis consumes a large amount of energy. Recently, it has been revealed that the nucleolus functions as a phase-separated compartment, temporarily sequestering misfolded proteins during heat shock, where the proteins are refolded in a heat shock protein (Hsp) 70-dependent manner during recovery [20,21,22,23].

In this study, we attempted to demonstrate how nucleolar actin dynamics are influenced by heat shock using a nuclear actin visualization probe, nAC-GFP, which is a GFP-labeled anti-actin chromobody with the nuclear localization signal (NLS). It was shown that nAC-GFP assembled in the nucleolus during heat shock at 42 °C and disassembled upon returning to 37 °C. This reversible disassembly was blocked by the Hsp70 inhibitor VER-155008. We hypothesized that actin plays a role in sequestering actin-binding proteins in the nucleolus to prevent the formation of their irreversible aggregation during heat shock. To predict which actin-binding proteins are sequestered in the nucleolus, we conducted bioinformatics analysis using integrated databases. It was shown that, among the 47 actin-binding proteins identified to localize in the nucleus, 10 displayed a higher proportion of intrinsically disordered regions (IDRs) compared to fibrillarin (FBL), a key nucleolar IDR protein [16]. Notably, two of these proteins, PDZ and LIM domain protein (PDLIM) 7 and TRIO-binding protein (TRIOBP), were highlighted because of their increased expression in response to heat shock. These results may provide clues for uncovering novel functions of actin in heat shock responses.

## 2. Materials and Methods

### 2.1. Cell Culture and Materials

Human cervical cancer HeLa (TKG, Miyagi, Japan) and human breast cancer MCF-7 (American Cell Type Culture Collection (ATCC), Manassas, VA, USA) cells were cultured in Dulbecco’s modified Eagle’s medium (Nissui Pharmaceutical, Tokyo, Japan) supplemented with 10% fetal bovine serum and 1% penicillin/streptomycin at 37 °C under 5% CO_2_. Heat shock was induced by incubating the cells at 42 °C under 5% CO_2_. VER-155008 and cycloheximide (CHX) were purchased from Selleck Chemicals (Tokyo, Japan) and Sigma-Aldrich (Tokyo, Japan), respectively.

### 2.2. Plasmid and Transfection

Cells were transfected with the expression vector nuclear Actin-chromobody^®^ plasmid obtained from ChromoTek (Planegg, Germany) using Lipofectamine 2000 transfection reagent (Invitrogen Thermo Fisher Scientific, Waltham, MA, USA) or PEI-MAX (Polysciences Inc., Warrington, PA, USA) according to the manufacturer’s instructions.

### 2.3. Retrovirus Infection

Retroviral infections were induced as previously described [15,24]. Briefly, to generate retroviruses encoding short hairpin RNA (shRNA) against human *p53*, the *p53* target sequence, 5′-GACTCCAGTGGTAATCTAC-3′, was cloned into a pSuper retro puro (Oligoengine, Seattle, WA, USA). Infected cells were selected using 1.5 µg/mL puromycin for 2–3 days.

### 2.4. Immunofluorescence and Live-Imaging

The cells were fixed with 4% paraformaldehyde (PFA) in phosphate-buffered saline (PBS) for 30 min at room temperature (RT) and permeabilized with 0.1% Triton X-100 for 15 min at RT for immunofluorescence. After blocking with 2% BAS in PBS, the cells were incubated with anti-cofilin rabbit monoclonal (D3F9: #5175; Cell Signaling Technology, Danvers, MA, USA), anti-fibrillarin rabbit monoclonal (C13C3: #2639; Cell Signaling Technology), and anti-NPM1 mouse monoclonal (0412: sc-47725; Santa Cruz Biotechnology, Santa Cruz, CA, USA) antibodies overnight at 4 °C, and washed three times with ice-cold PBS. The cells were incubated overnight at 4 °C or 30 min at room temperature with Alexa Fluor 546-conjugated goat anti-rabbit IgG (Invitrogen, Waltham, MA, USA), Alexa Fluor 647-conjugated goat anti-mouse IgG (Invitrogen), together with or without Alexa Fluor 546-conjugated Phalloidin (Invitrogen) to stain F-actin. Nuclei were then stained with 4′,6-Diamidino-2-phenylindole (DAPI; Vector Laboratories, Newark, CA, USA). Images were acquired using a confocal microscope (A1R HD25; Nikon, Tokyo, Japan) and analyzed using the Fiji software (version 1.54f).

Alternatively, the cells were monitored using an A1R HD25 confocal microscope equipped with a live cell imaging system, capturing time-lapse DIC and fluorescence images every 3 min over a period of 60 min.

### 2.5. Bioinformatic Analysis

Lists of actin-binding and nucleolus-localized proteins were obtained from UniProtKB “https://www.uniprot.org (2 December 2024)” and Harmonizone 3.0 “https://maayanlab.cloud/Harmonizome/ (2 December 2024)” [25], respectively. IDRs within the proteins were predicted using IUPred2A “https://iupred2a.elte.hu (2 December 2024)”. The predictive type of option was performed with “IUPred2 long disorder”. Quantitative proteome data of human melanoma M14 cells donated to Gene Expression Omnibus (GEO) were used to refer the proteome data (LS-MS/MS) from heat shock treatment [26]. The 3D structure of the protein was predicted using AlphaFold2 and AlphaFold-Multimer [27,28]. Protein–protein interaction analysis was performed using STRING network analysis (version 12.0) “https://string-db.org (2 December 2024)”. The minimum required interaction score was set to medium confidence (0.400) and the maximum number of interactors to show was set to no more than 15.

## 3. Results

**Figure 1:**
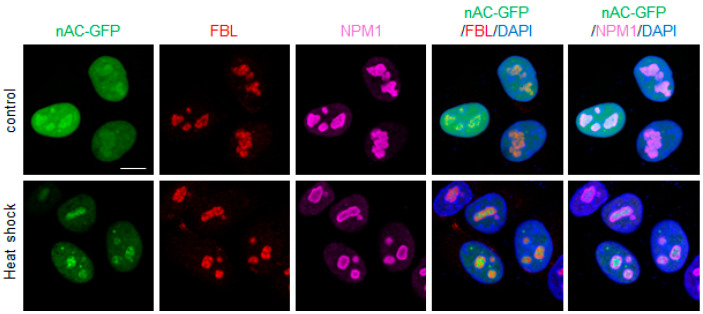
**Heat shock induces nAC-GFP assembly in the nucleolus.** HeLa cells were transfected with the nAC-GFP expression vector and treated with heat shock (42 °C for 60 min). Confocal images of nAC-GFP (green), FBL (red), NPM1 (magenta), and DNA (blue). Scale bar = 10 μm.

Tumor suppressor p53 plays a pivotal role in cellular responses to various stressors, including DNA damage and heat shock [29,30]. In a previous study, p53 deficiency was shown to promote the formation of nuclear actin filaments in response to DNA damage [15]. Given that heat shock induces nuclear actin assembly and activates p53, this study aimed to determine the role of p53 in actin assembly following heat shock treatment. To investigate this, human breast cancer MCF-7 cells bearing wild-type (WT) p53 with or without p53 knockdown using shRNA were used. The detection of nuclear actin filaments using fluorescent phalloidin is challenging because the level of F-actin is much lower in the nucleus than in the cytoplasm, and the molecular structure of nuclear actin filaments is potentially distinct from that of cytoplasmic actin filaments [15,31]. The cells were transfected with an expression vector for nAC-GFP, enabling the visualization of nuclear actin dynamics. In unstimulated control cells, nAC-GFP was distributed throughout the nucleoplasm, with certain regions showing higher concentrations of nAC-GFP (Supplementary Figure S1). These regions were likely nucleoli, as suggested by their structure, and were outlined by the surrounding heterochromatin. After 60 min of heat exposure at 42 °C, nAC-GFP assembly was observed in the presumed nucleolus. The distribution of nAC-GFP in response to heat shock was not influenced by p53 knockdown, suggesting that heat shock promotes actin assembly in the nucleolus but not in the nucleoplasm in a p53-independent manner. To further investigate this, HeLa cervical cancer cells expressing viral oncoprotein E6 [32], which inactivates p53, were used. HeLa cells, transfected with the nAC-GFP expression vector, were incubated at 42 °C for 60 min and then fixed for immunostaining with antibodies against FBL and nucleophosmin 1 (NPM1), which are well-established markers of the nucleolus. In heat shock-treated cells, nAC-GFP assembly was localized to the nucleus, where FBL and NPM1 were present (Figure 1). Thus, actin assembly appears to occur specifically in the nucleolar regions upon heat shock. These findings suggest that p53 is dispensable for heat shock-induced nucleolar actin assembly and highlight a potential p53-independent mechanism for regulating nucleolar dynamics under heat shock conditions.

It was previously demonstrated that nAC-GFP induces structural changes in nuclear actin filaments in p53-depleted cells, leading to the formation of phalloidin-stainable structures under DNA damage stress [15]. To further investigate the nature of nAC-GFP assembly under heat shock, cells transfected with the nAC-GFP expression vector were subjected to heat shock and stained with phalloidin. Unlike the nuclear actin filaments formed in response to DNA damage-induced stress, the assembled nAC-GFP in the nucleolus after heat shock treatment was not stained with phalloidin (Supplementary Figure S2a). In parallel, it has been reported that heat shock induces the formation of actin–cofilin rods in the nucleoplasm of mouse fibroblast C3H-2K cells although these structures are not observed in the nucleoli [11,33,34]. To investigate whether actin assembly in the nucleoli of HeLa cells after heat shock were associated with cofilin, immunostaining was performed using an anti-cofilin antibody. Cofilin was distributed throughout both the cytoplasm and nucleus, but its levels in the nucleolus were low, even under heat shock conditions (Supplementary Figure S2b). It was also shown that cofilin did not colocalize with the assembled nAC-GFP in the nucleolus, suggesting that the actin assembly in the nucleolus formed in response to heat shock were distinct from actin–cofilin rods.

Recently, it was revealed that heat shock causes the accumulation of misfolded proteins, some originating from defective ribosomal products (DRiPs), in the nucleolus, where they form structures known as amyloid bodies (A-bodies) [20,35]. The formation of A-bodies disassembles in an Hsp70-dependent manner during recovery from heat shock [21]. Since the shape of the nAC-GFP assembly in the nucleolus resembled that of A-bodies, we hypothesized that actin might be present in the A-bodies.

**Figure 2:**
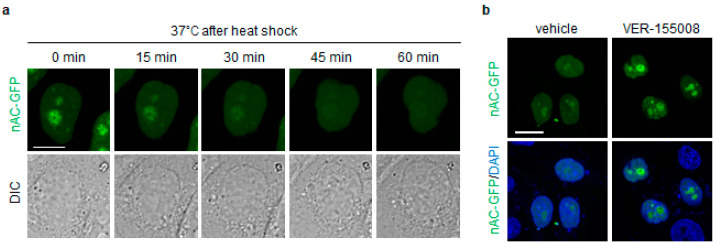
**The reversibility of heat shock-induced nAC-GFP assembly is suppressed by the inhibition of Hsp70.** HeLa cells were transfected with nAC-GFP expression vector and then treated with heat shock (42 °C for 60 min), followed by recovery at 37 °C. (**a**) The nAC-GFP assembly was observed over 60 min by confocal microscopy with time-lapse imaging. Confocal images of nAC-GFP (green) and DIC (grey) are shown. Scale bar = 10 μm. (**b**) Hsp70 inhibitor VER-155008 (VER; 50 μM) was added to the culture medium immediately before recovery at 37 °C. After 90 min of incubation at 37 °C, the cells were fixed. Confocal images of nAC-GFP (green) and DAPI (blue) are shown. Scale bar = 20 μm.

To determine whether heat shock-induced nAC-GFP assembly is disassembled in an Hsp70-dependent manner during recovery, we used the Hsp70 inhibitor VER-155008. We observed that nAC-GFP assembly in the nucleolus gradually disappeared within 60 min after treating cells at 42 °C for 60 min and then returning them to 37 °C (Figure 2a, Supplementary Videos S1 and S2), indicating that the nAC-GFP assembly was reversible. We also pre-treated the cells with VER-155008 immediately before returning them to 37 °C, incubated them for 90 min, and then fixed them. nAC-GFP assembly persisted in the nucleoli of cells treated with VER-155008 (Figure 2b), suggesting that the disassembly of nAC-GFP in the nucleolus during recovery relies on Hsp70. The finding that Hsp70 inhibition prevented the reversibility of heat shock-induced nAC-GFP assemblies strongly suggests that these structures contain misfolded nAC-GFP.

**Figure 3:**
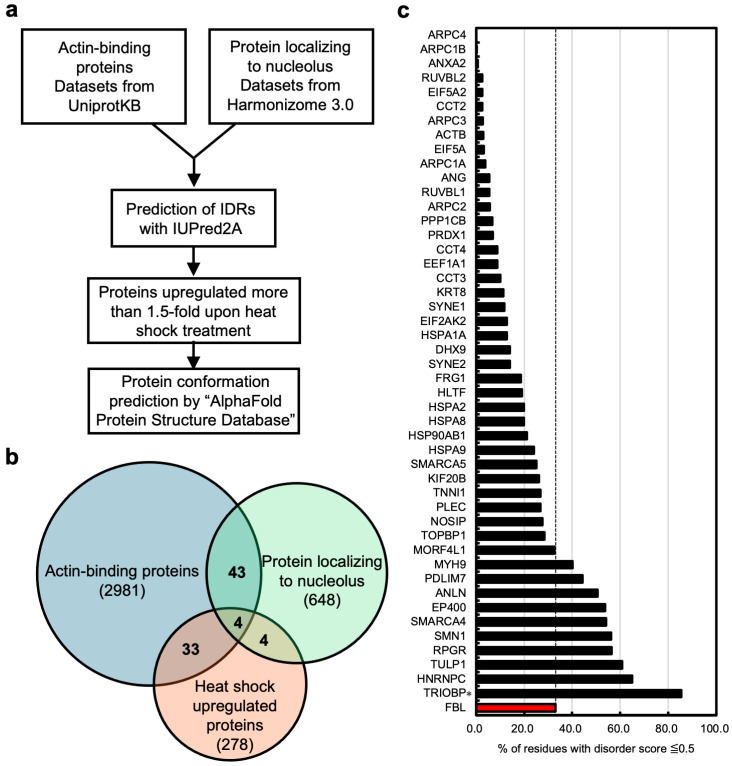
**Prediction of actin-binding proteins localized to the nucleolus under heat shock conditions.** (**a**) The workflow of bioinformatic analysis in this study. The datasets of actin-binding proteins and nucleolus localized proteins were filtered from UniProtKB and a nucleolus gene set of LOCATE-curated protein localization annotations (Harmonizome 3.0), respectively. In this study, proteomic data (LS-MS/MS) of human melanoma M14 cells under heat shock conditions were obtained from gene expression omnibus (GEO) and re-profiled. Protein 3D structures were predicted using AlphaFold2and AlphaFold-Multimer. (**b**) The Venn diagram illustrates the overlap of actin-binding proteins (2981 proteins, blue) and nucleolus-localized proteins (648 proteins, green). From this set, the gene names of 47 proteins common to both actin-binding and nucleolus-localized proteins were identified. Additionally, the Venn diagram includes proteins whose expression levels increased by more than 1.5-fold upon heat shock treatment (278 proteins, orange). (**c**) The IDRs in 47 proteins extracted by (**b**) were analyzed by using IUPred2A. The high disorder regions in this study were defined as amino acid region in which disorder score is higher than 0.5. The X axis indicates the percentage of high disorder regions to the entire amino acid sequence. FBL is not classified as an actin-binding protein; but it was employed as a nucleolar IDR protein indicator. Note that TRIOBP * indicates the longest isoform TRIOBP-6.

Nevertheless, previous studies have demonstrated that heat shock influences nuclear actin dynamics [11,33,34,36,37], raising the possibility that actin may also sequester actin-binding proteins in the nucleolus to prevent their irreversible aggregation. Given the challenges in elucidating the heat shock response using nAC-GFP, we performed bioinformatics analysis, following the workflow shown in Figure 3a, to predict actin-binding proteins. First, we identified actin-binding proteins that localized to the nucleolus based on datasets from UniProt and Harmonizome 3.0 [25]. There were 2981 actin-binding proteins and 648 nucleolus-localized proteins, of which 47 proteins were common to both datasets (Figure 3b). Assuming that actin-binding proteins are sequestered in the nucleolus to prevent irreversible aggregation under heat shock, proteins capable of forming LLPS-driven droplets are considered potential candidates for sequestration into the nucleolus during heat shock. We analyzed all 47 proteins for IDRs using IUPred2A. TRIOBP exists in six isoforms [38], and the longest isoform, TRIOBP-6, was analyzed. The 10 proteins among the 47 proteins exhibited a greater proportion of IDRs than FBL (Figure 3c, Table 1, and Supplementary Figure S3), a major nucleolar protein recognized as an IDR protein [39]. Therefore, we considered that these 10 proteins are highly likely to be sequestered in the nucleolus in response to heat shock.

**Figure 4:**
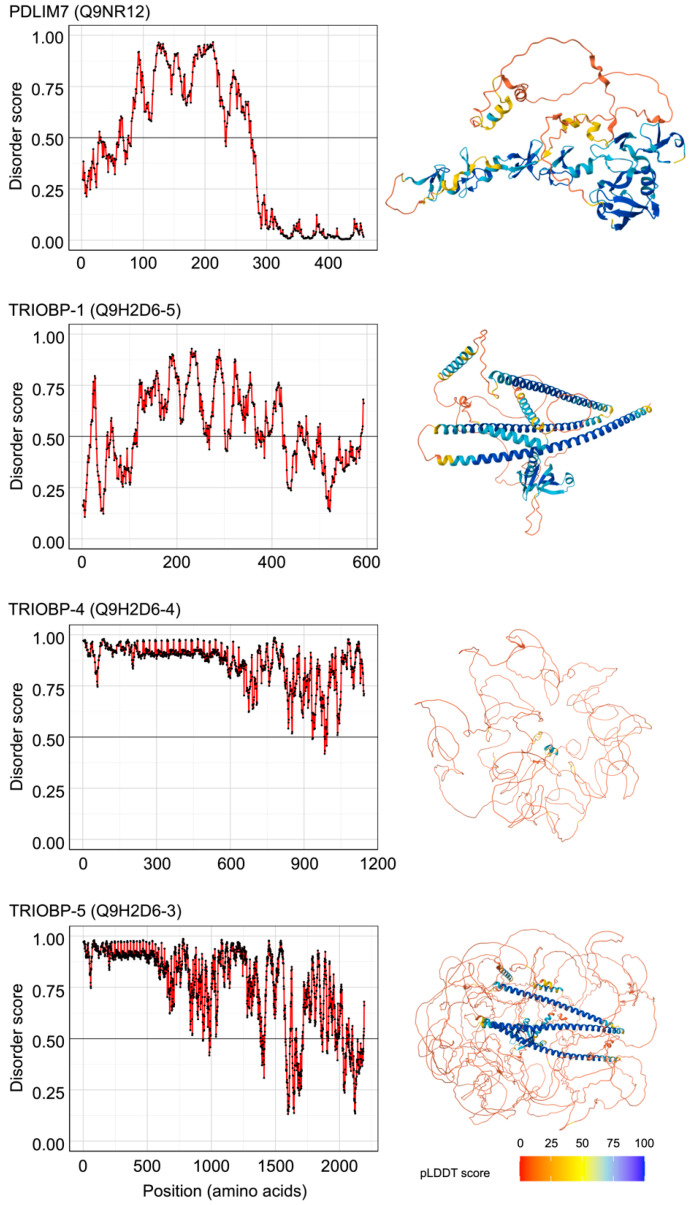
Prediction of IDRs and three-dimensional structures of PDLIM7 and TRIOBP. The left graph indicates plots the accurate IDR predictions for PDLIM7, TRIOBP-1, TRIOBP-4, and TRIOBP-5, as determined by IUPred2A. The X- and Y-axes represent the position of the peptide chain in the protein and the disorder score, which indicates the degree of IDR, respectively. Regions with a disorder score of 0.5 or above indicate a high probability of being disordered regions. The right panels show the three-dimensional structure of the proteins predicted using AlphaFold2 and AlphaFold-Multimer. The color scale shows the predicted local distance difference test (pLDDT) score, which is a measure of prediction accuracy for each amino acid residue.

We also speculated that actin-binding proteins with increased levels in response to heat shock have a higher potential to translocate to the nucleolus, and referred to the proteomics database comparing heat shock-treated and non-treated human melanoma M14 cells [26]. The levels of these four proteins were at least 1.5 times higher in heat-shock cells than in untreated cells (Figure 3b, Supplementary Table S1). Among them, PDLIM7 and TRIOBP ranked within the top 10 proteins, with a higher proportion of IDRs than FBL (Table 1). Additionally, three-dimensional structural predictions using AlphaFold suggested that both PDLIM7 and TRIOBP are IDR proteins (Figure 4). In summary, through comprehensive bioinformatics analysis, we identified PDLIM7 and TRIOBP as proteins with high IDR content and increased level in response to heat shock. These proteins were considered strong candidates for sequestration into the nucleolus by actin under heat shock stress.

## 4. Discussion

The primary objective of this study was to investigate nuclear actin dynamics in response to heat shock using nAC-GFP. Through our experiments, we showed that heat shock induces nAC-GFP assembly contains misfolded proteins, and analyzing these proteins using nAC-GFP is challenging, we performed a bioinformatics analysis to predict which actin-binding proteins were sequestered in the nucleolus under heat shock conditions. As a result, we identified 10 actin-binding proteins with nucleolar localization that were identified as having high IDR content. Notably, PDLIM7 and TRIOBP were suggested to be particularly important, as their expression levels increased significantly upon heat shock.

PDLIM7 is an adaptor protein with an N-terminal PDZ domain and three C-terminal LIM domains connected by a long intrinsically disordered linker [40], and it binds to actin filaments via the PDZ domain. Using STRING network analysis, we investigated the relationship between PDLIM7 and five heat shock proteins, which were included in the 47 identified actin-binding proteins that identified to localize in the nucleolus (Supplementary Table S2). Our analysis indicated that PDLIM7 interacts with the Hsp70 family members HSPA2 (heat shock protein family A member 2) and HSPA8 (Hsc70, heat shock cognate 70) through cochaperone BAG3 (Bcl-2-associated athanogene 3), which binds to other heat shock proteins, including another Hsp 70 family member HSPA1B (Supplementary Figure S4). BAG3, a multifunctional and ubiquitous protein cooperates with Hsp70 to sequester misfolded proteins [41,42], including DRiPs, into aggresomes, where they are targeted for degradation via autophagy, thereby supporting cell survival and normal function [43]. Although BAG3 is not classified as a nucleolar protein in the Harmonizome 3.0 dataset, considering that heat shock stress induces BAG3 expression and translocation to the nucleus [44], the BAG3-Hsp70 complex may also function in the nucleolus. Further investigation of the interactions between BAG3, Hsp70 family proteins, and PDLIM7 in the nucleolus could provide new insights into the role of nucleolar actin in heat shock response.

TRIOBP plays a crucial role in stabilizing actin filaments and exists in six isoforms generated through distinct transcription start sites and alternative splicing [38]. Among these, the longest isoform is TRIOBP-6, followed by the slightly shorter TRIOBP-5, and the most extensively studied isoforms are TRIOBP-1 and TRIOBP-4. TRIOBP-1 is transcribed from the 3′ end of the TRIOBP gene and is widely expressed across many tissues, indicating its involvement in a broad range of cellular functions. In contrast, TRIOBP-4 is transcribed from the 5′ end of the gene, without sharing exons with TRIOBP-1. TRIOBP-4 is primarily expressed in the inner ear and retina, although it has also been observed in HeLa cells [38,45]. Additionally, TRIOBP-5 has been reported to be highly expressed in pancreatic cancer cells [38,46]; however, its expression in HeLa cells remains unclear. Figure 3c shows the results of IDR analysis for TRIOBP-6, which is the longest isoform. Figure 4 presents the IDR predictions for TRIOBP-1, TRIOBP-4, and TRIOBP-5 using IUPred2. TRIOBP-1 has a tendency to self-associate, leading to the formation of insoluble aggregates, whereas TRIOBP-4 is a fully disordered protein [38]. Additionally, TRIOBP-5 is thought to have aggregation properties at its N-terminus, similar to those of TRIOBP-1, whereas its C-terminus exhibits properties of forming liquid droplets similar to TRIOBP-4. Although the STRING network did not show any association between TRIOBP and the five heat shock proteins (Supplementary Figure S4), cellular analysis of the relationship between the structure of TRIOBP isoforms and their association with actin under heat shock stress may provide insights into novel cellular response mechanisms within the heat shock response. To gain a deeper understanding of how actin regulates nucleolar functions and the role of actin dynamics in the heat shock response, it is essential to further investigate whether candidate proteins beyond PDLIM7 and TRIOBP, which were selected based on the possibility that actin assembly may form in the nucleolus under heat shock, are necessary for the formation of these actin assembly, as well as their importance in the heat shock response.

Many actin-binding proteins have been reported to contain IDRs within their structures [47]. In addition to the nucleoli, focal adhesions, stress granules, and transcriptional complexes are regulated by LLPS [48], and dysregulation of LLPS is linked to the onset and progression of diseases such as cancer and neurodegenerative disorders [49,50,51]. Understanding the roles of actin and its binding proteins with IDRs in response to heat shock and other stress stimuli may provide insights into the mechanisms underlying these diseases.

## Figures and Tables

**Table 1:** List of actin-binding proteins with nucleolar localization that were identified as having high IDR content.

Protein Symbol	PROTEIN NAME	Primary Accession ID	Amino Acids	% of Residues with Disorder Score ≥ 0.5	Ratio (HS/NHS)
MYH9	Myosin Heavy Chain 9	P35579	1960	40.2	1.24
PDLIM7	PDZ And LIM Domain 7	Q9NR12	457	44.2	169.23
ANLN	Anillin, Actin Binding Protein	Q9NQW6	1124	50.6	N.D.
EP400	E1A Binding Protein P400	Q96L91	3159	53.8	N.D.
SMARCA4	SWI/SNF Related, Matrix Associated, Actin Dependent Regulator of Chromatin, Subfamily A, Member 4	P51532	1647	54.2	N.D.
SMN1	Survival of Motor Neuron 1, Telomeric	Q16637	294	56.1	N.D.
RPGR	Retinitis Pigmentosa GTPase Regulator	Q92834	1020	56.5	N.D.
TULP1	TUB Like Protein 1	O00294	542	60.9	N.D.
HNRNPC	Heterogeneous Nuclear Ribonucleoprotein C	P07910	306	65.0	0.71
TRIOBP	TRIO And F-Actin Binding Protein	Q9H2D6	2365	85.5	205.11

Among the actin-binding proteins in the nucleolus, 10 proteins were listed with a higher percentage of residues with a higher disorder score than FBL (i.e., ≥0.5). Protein level under heat shock (HS) to protein level non-heat shock (NHS) based on proteomic data (Figure 3a). “N.D.” indicates that no data are available.

## Data Availability

The original contributions presented in the study are included in the article/Supplementary Materials, further inquiries can be directed to the corresponding author.

## Supplementary Materials

The following supporting information can be downloaded at: https://www.mdpi.com/article/10.3390/genes15121580/s1. Figure S1: Heat shock induces the assembly of nAC-GFP in the nucleolus in a p53-independent manner; Figure S2: Heat shock-induced nAcGFP assemblies can not be stained by phalloidin; Figure S3: Analysis of IDRs in actin-binding proteins identified to localize in the nucleolus; Figure S4: STRING analysis showing protein-protein interactions between PDLIM7, TRIOBP, and heat shock proteins; Table S1: List of actin-binding proteins identified to localize in the nucleolus with increased expression levels following heat shock treatment; Table S2: List of actin-binding heat shock proteins that localized to the nucleolus; File S1: Torii heatshock proteo analysis S1; Video S1: HeLa nAC-GFP 42dC 1 h-37dC 1 h Live GFP; Video S2: HeLa nAC-GFP 42dC 1 h-37dC 1 h Live DIC.
