# Supplementary material for: Bioinformatic Analysis of Actin-Binding Proteins in the Nucleolus During Heat Shock
Source: Genes (Basel). 2024 Dec 9;15(12):1580. doi: 10.3390/genes15121580 (PMC11675300; doi:10.3390/genes15121580)
Supplement: Supplementary file 1 [file genes-15-01580-s001.zip › Second Revise supplementary information Genes.pdf]

Bioinformatic Analysis of Actin-Binding Proteins in the Nucleolus During Heat Shock

Shinya Taniguchi[1*], Takeru Torii[1*], Toshiyuki Goto[2], Kohei Takeuchi[1], Rine Katsumi[1], Mako Sumida[1], Sunmin Lee[1], Wataru Sugimoto[1], Masaya Gessho[1], Katsuhiko Itoh[1], Hiroaki Hirata[3], Junji Kawakami[1], Daisuke Miyoshi[1], Keiko Kawauchi[1†*]

Supplementary Information Contents:

Supplementary Figures S1 - S4

Supplementary Tables S1 - S2

Supplementary material and methods

Reference

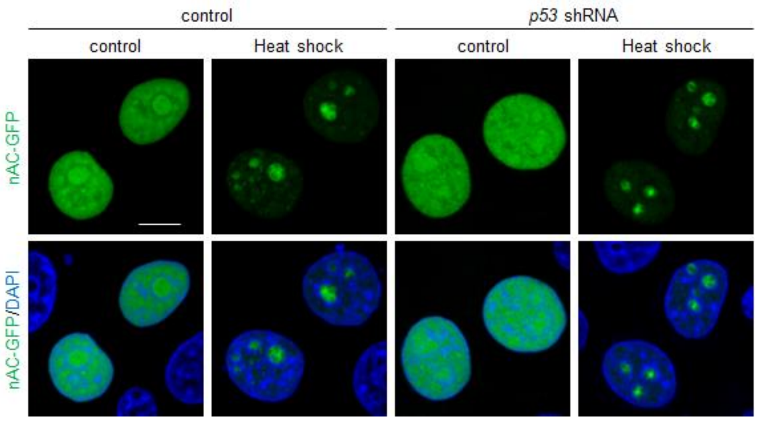

Supplementary Figure S1. Heat shock induces the assembly of nAC-GFP in the nucleolus in a p53-independent manner.

MCF-7 cells expressing control or *p53* shRNA were transfected with nAC-GFP expression vector and then treated with heat shock (42°C for 60 min). Confocal images of nAC-GFP (green) and DNA stained using DAPI (blue) are shown. Scale bar = 10 μm.

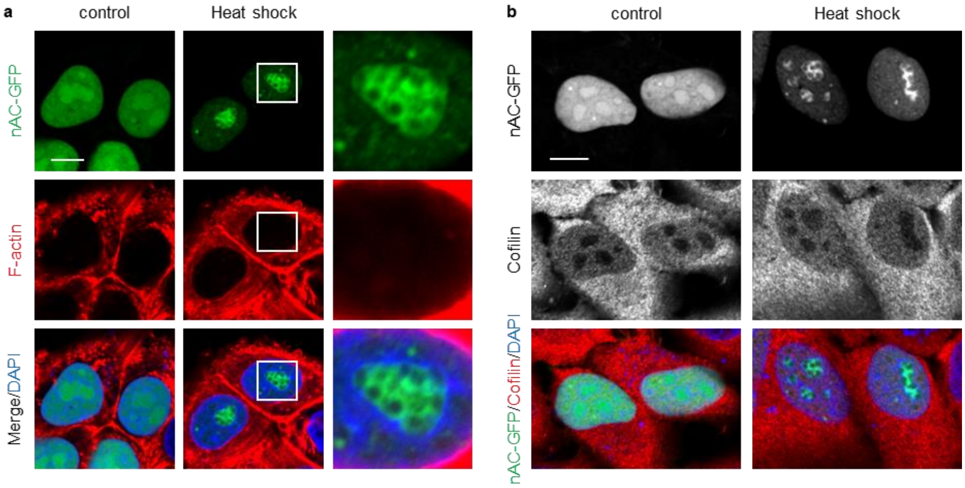

Supplementary Figure S2. Heat shock-induced nAcGFP assemblies can not be stained by phalloidin. HeLa cells were transfected with nAC-GFP expression vector and then treated with or without heat shock (42°C for 60 min). (**a**) Confocal images of nAC-GFP (green), F-actin stained with phalloidin (red), and DNA stained with DAPI (blue). The right panel shows magnified images indicated by squares. The red fluorescence intensity in the right panels increased. (**b**) Confocal images of nAC-GFP (grey/green), cofilin (grey/red), and DNA stained with DAPI (blue). Scale bar = 10 μm.

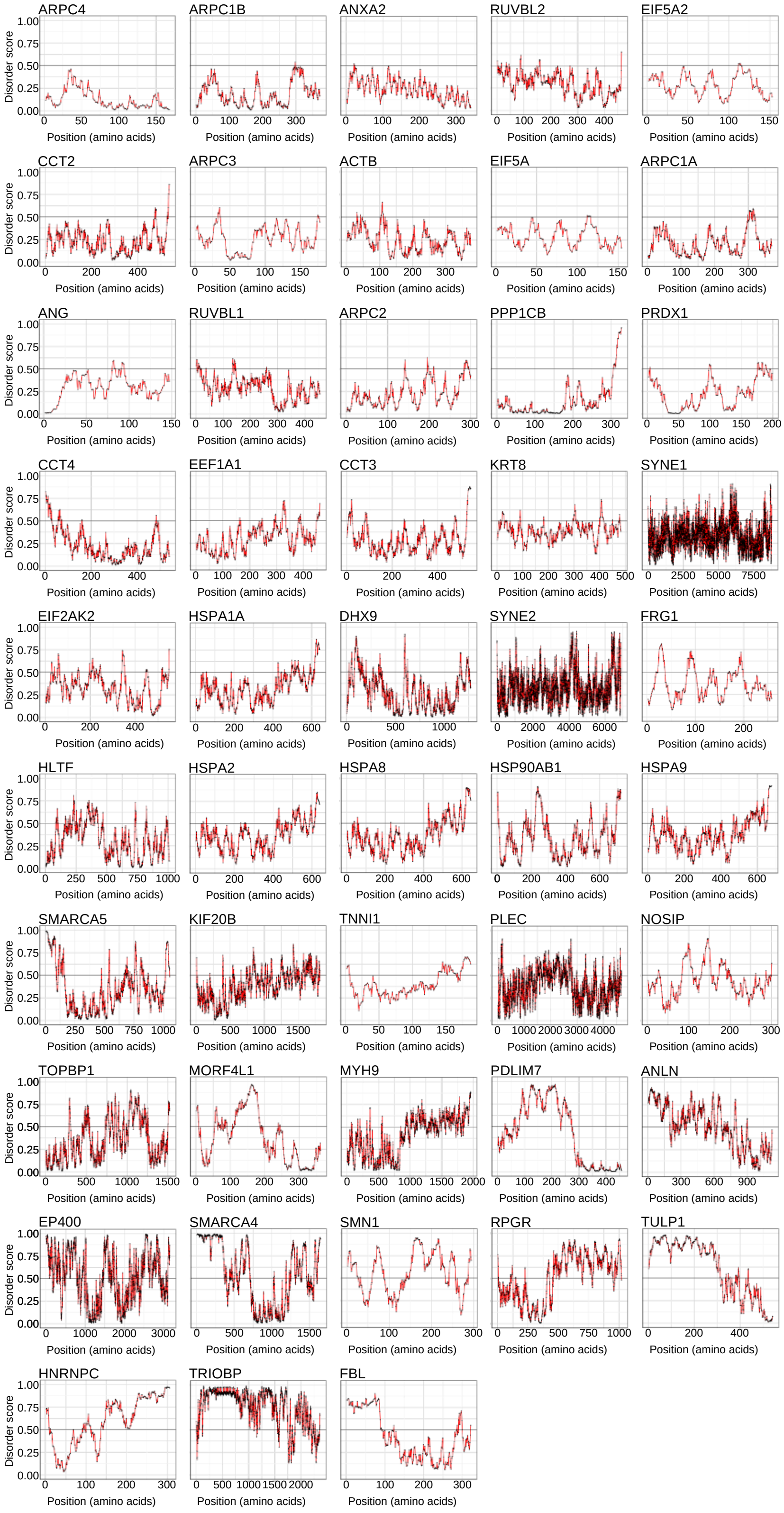

Supplementary Figure S3. Analysis of IDRs in actin-binding proteins identified to localize in the nucleolus.

Dataset analysis identified 47 proteins for which both actin-binding and nucleolus localization were confirmed. The IDRs of these proteins were analyzed using IUPred2A. The X- and Y-axes indicate the amino acid positions and disorder scores, respectively. The last graph shows result for fibrillarin (FBL), a nucleolus-localized protein, are shown for reference. The high-disorder regions are defined as amino acid regions with disorder scores higher than 0.5.

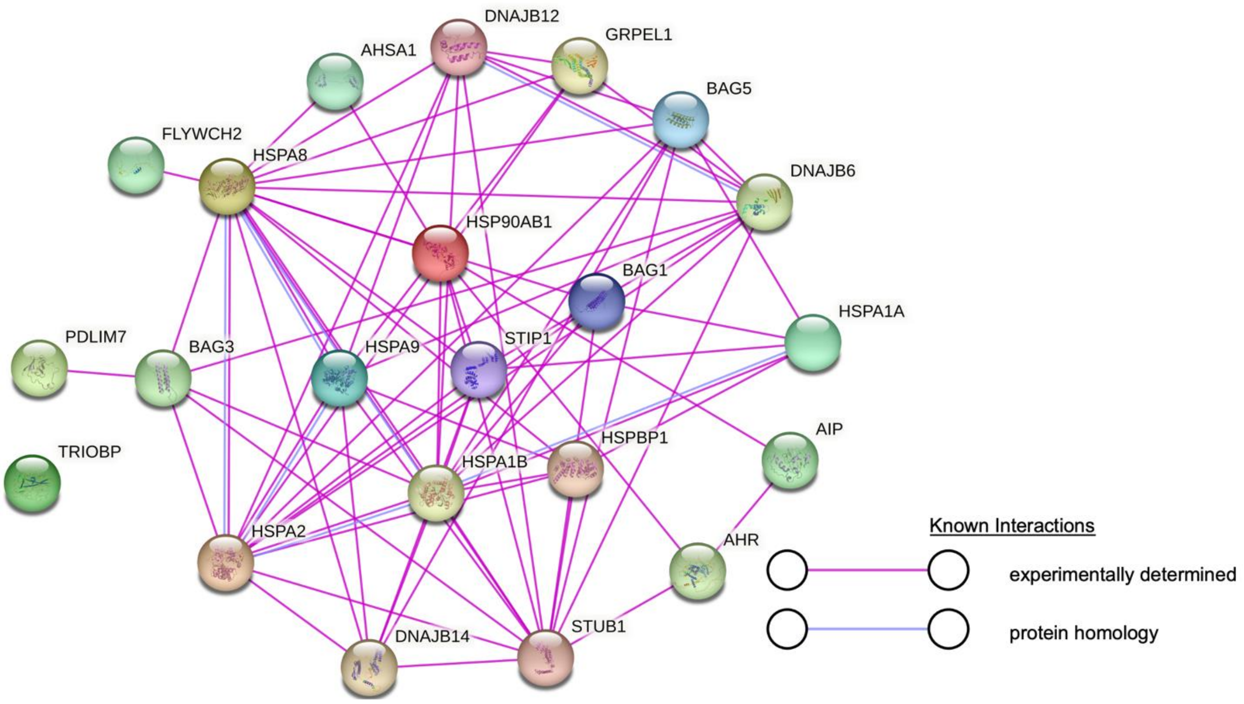

Supplementary Figure S4. STRING analysis showing protein-protein interactions between PDLIM7, TRIOBP, and heat shock proteins.

Proteins interacting with PDLIM7, TRIOBP, and heat shock proteins among the actin-binding 47 proteins that identified to localize in the nucleolus were predicted using STRING analysis. The minimum required interaction score and the maximum number of interactors were set to medium confidence (0.400) and 15, respectively. Each node shows the query proteins or the first shell of the interactors. Empty and filled nodes indicate undetermined and determined/predicted 3D structures, respectively.

Supplementary Table S1. List of actin-binding proteins identified to localize in the nucleolus with increased expression levels following heat shock treatment.

Protein symbol	Protein name	Ratio (HS/NHS)
ANXA2	Annexin A2	1.59
EEF1A1	Eukaryotic translation elongation factor 1 alpha 1	1.26
PDLIM7	PDZ and LIM domain protein 7	169.23
TRIOBP	TRIO binding protein	205.11

The expression level of heat shock (HS) versus non-heat shock (NHS) in listed proteins were referred from proteomic data (Fig. 3a; [1]). From Fig. 3b, among the 47 actin-binding proteins identified as localized to the nucleolus, four showed increased expression levels following heat shock treatment. All listed proteins, except for EEF1A1, were expressed in HeLa cells, according to the Human Protein Atlas database.

Supplementary Table S2. List of actin-binding heat shock proteins that localized to the nucleolus

Protein symbol	Protein name	Ratio (HS/NHS)
HSPA1A	Heat shock protein family A (Hsp70) member 1A	15.62
HSPA2	Heat shock protein family A (Hsp70) member 2	1.13
HSPA8	Heat shock protein family A (Hsp70) member 8	1.30
HSPA9	Heat shock protein family A (Hsp70) member 9	1.07
HSP90AB1	Heat shock protein 90 alpha family class B member 1	1.24

The expression level of heat shock (HS) versus non-heat shock (NHS) in listed proteins were referred form proteomic data (Fig. 3a; [1]). From Fig. 3b, of the 47 actin-binding proteins that identified localized to the nucleolus, five had known as heat shock proteins were extracted. All listed proteins, except for HAPA1A, were expressed in HeLa cells, according to the Human Protein Atlas database.

Reference

[1] W. Miao, Y.Y. Yang, and Y. Wang, Quantitative Proteomic Analysis Revealed Broad Roles of N(6)-Methyladenosine in Heat Shock Response. J Proteome Res 20 (2021) 3611-3620.
